# The Effect of Gout on Thyroid Cancer Incidence: A Nested Case-Control Study Using a National Health Screening Cohort

**DOI:** 10.3390/jpm12060887

**Published:** 2022-05-27

**Authors:** So Young Kim, Dae Myoung Yoo, Mi Jung Kwon, Ji Hee Kim, Joo-Hee Kim, Woo Jin Bang, Sung Kyun Kim, Hyo Geun Choi

**Affiliations:** 1 Bundang CHA Medical Center, Department of Otorhinolaryngology-Head and Neck Surgery, CHA University, Seongnam 13488, Korea; sossi81@hanmail.net; 2 Hallym Data Science Laboratory, Hallym University College of Medicine, Anyang 14068, Korea; ydm1285@naver.com; 3 Department of Pathology, Hallym Sacred Heart Hospital, Hallym University College of Medicine, Anyang 14068, Korea; mulank@hanmail.net; 4 Department of Neurosurgery, Hallym University College of Medicine, Anyang 14068, Korea; kimjihee.ns@gmail.com; 5 Department of Medicine, Division of Pulmonary, Allergy, and Critical Care Medicine, Hallym Sacred Heart Hospital, Hallym University College of Medicine, Anyang 14068, Korea; luxjhee@gmail.com; 6 Department of Urology, Hallym Sacred Heart Hospital, Hallym University College of Medicine, Anyang 14068, Korea; yybbang@gmail.com; 7 Department of Otorhinolaryngology-Head and Neck Surgery, Hallym University College of Medicine, Anyang 14068, Korea; madein811022@gmail.com

**Keywords:** gout, thyroid cancer, risk factors, cohort studies, epidemiology

## Abstract

In this study, we investigated the risk of thyroid cancer in patients with gout. Participants ≥40 years old in the Korean National Health Insurance Service–Health Screening Cohort were investigated. A total of 5844 patients with thyroid cancer were paired with 23,376 comparison participants (no history of thyroid cancer) to build the nested case–control study. A previous history of gout was collected from both the thyroid cancer and comparison participants. The relationship of thyroid cancer with a prior history of gout was analyzed using a conditional logistic regression model. The rate of gout was higher in the patients with thyroid cancer than in the comparison group. In the total study population, the relationship of thyroid cancer with a prior history of gout was not statistically evident. A previous history of gout was related to an increased risk of gout in the <60 years old, normal weight, abnormal blood pressure, and CCI score = 0 groups. The occurrence of thyroid cancer was not associated with a prior history of gout in the general adult population. However, a prior history of gout was related to an elevated risk of thyroid cancer in middle-aged and healthy populations without comorbidities.

## 1. Introduction

Thyroid cancer is a common malignancy with a rising incidence worldwide [1]. In Korea, the diagnosis of thyroid cancer has grown 15-fold from 1993 to 2011 [2]. Although the pathophysiologic cause of thyroid cancer is still controversial, multiple factors aggravate the risk of thyroid cancer [3]. A radiation overdose, the female sex, an iodine intake deficit, and a family history of thyroid cancer are known to increase the risk of thyroid cancer [4]. In addition, metabolic disorders, including obesity and metabolic syndrome, are associated with a higher incidence of thyroid cancer (hazard ratio = 1.39, 95% confidence intervals (95% CI) = 1.33–1.44) [5]. Moreover, an abnormal thyroid function or diseases are associated with an increased incidence of thyroid cancer [6]. A nationwide cohort study in Denmark presented standardized incidence ratios for thyroid cancer as high as 2.00–6.02-fold in patients with hyperthyroidism, goiter, and adenoma [6].

Gout is characterized by the accumulation of monosodium urate crystals owing to high urate concentrations (hyperuricemia) [7]. Gout is a common chronic disease with a prevalence as high as 1–6.8% and an incidence of 0.58–2.89 per 1000 persons/year worldwide [8]. The status of hyperuricemia and flares of gout can be precipitated by the inflammatory activation of the NOD-, LRR- and pyrin domain-containing protein 3 (NLRP3) inflammasomes and the secretion of interleukin 1β [7]. In addition to inflammatory conditions, gout is related to metabolic derangements such as metabolic syndrome [9]. Furthermore, a few prior studies have suggested a relationship between gout and thyroid abnormalities, with a few conflicting results [10]. Thyroid cancer is associated with benign thyroid diseases, including hyperthyroidism, thyroiditis, and autoimmune thyroid diseases [11]. In addition, both thyroid cancer and gout are associated with metabolic disorders. Thus, it can be supposed that patients with gout may have a higher susceptibility to thyroid cancer.

We hypothesized that patients with gout may have a higher susceptibility to thyroid cancer. To test this hypothesis, patients with thyroid cancer were analyzed for a previous history of gout. Although previous studies have pointed to the relationship of thyroid dysfunctions with gout, there is a lack of knowledge on the association of thyroid cancer with gout. As both thyroid cancer and gout are related to comorbidities such as cardiovascular diseases and metabolic diseases, these factors were considered to be covariates in this study.

## 2. Materials and Methods

### 2.1. Study Population

We used the Korean National Health Insurance Service–Health Screening Cohort data for this study. A comprehensive explanation for this cohort is provided elsewhere [12]. This study was approved by the ethics committee of Hallym University (IRB No: 2019-10-023) following the guidelines of the IRB.

### 2.2. Participant Selection

**Figure 1:**
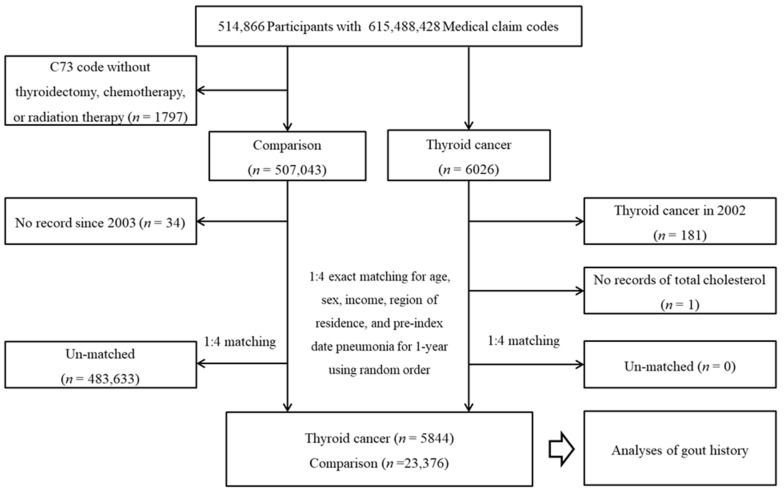
A schematic illustration of the participant selection process used in the present study. Of a total of 514,866 participants, 5844 thyroid cancer participants and 23,376 comparison participants were matched at a 1:4 ratio according to age, sex, income, and region of residence.

From a total of 514,866 individuals with 615,488,428 medical claim codes, thyroid cancer participants were selected according to the definition in our study (*n* = 6026). Among the thyroid cancer group, thyroid cancer participants who were diagnosed in 2002 (washout period, *n* = 181) or had no record of total cholesterol (*n* = 1) were removed. Among the other participants, participants who were diagnosed with C73 (malignant neoplasm of the thyroid gland) using ICD-10 codes without thyroidectomy, chemotherapy, or radiation therapy were removed (*n* = 1797). The rest of the patients were selected as a comparison group (*n* = 507,043). The participants who died before 2003 or had no records since 2003 were excluded from the comparison group (*n* = 34). The thyroid cancer participants and comparison participants were paired at a ratio of 1:4 for age, sex, income, and region of residence. The comparison participants were chosen in a random order. The index date of the thyroid cancer participants was defined as the index date of the paired comparison participants. Thus, each thyroid cancer participant with a paired comparison participant had an identical index date. A total of 483,633 comparison participants were removed during matching. Finally, 5844 thyroid cancer participants were paired with 23,376 comparison participants (Figure 1).

### 2.3. Thyroid Cancer (Outcome)

Thyroid cancer was defined using ICD-10 codes (C73, Malignant neoplasm of thyroid gland). Among them, we selected the participants who underwent thyroid surgery (claim codes: P4551, P4552, P4553, P4554, and P4561), chemotherapy, or radiation therapy, following our previous studies [13].

### 2.4. Gout (Exposure)

Gout was defined as visiting a clinic or hospital with a diagnosis of gout (ICD-10: M10, gout) ≥2 times. These methods were modified from a previous study [13].

### 2.5. Covariates

The 10 age groups were classified into 5-year intervals. The 5 income groups were defined (class 1 (poorest)–5 (richest)). The urban and rural areas were grouped [14]. Data on tobacco smoking, alcohol consumption, and obesity were collected [15]. Total cholesterol (mg/dL), systolic blood pressure (SBP, mmHg), diastolic blood pressure (DBP, mmHg), and fasting blood glucose (mg/dL) were measured. The Charlson Comorbidity Index (CCI) was used to measure the medical histories of 17 patients without thyroid cancer.

### 2.6. Statistical Analyses

The demographic features of the thyroid cancer groups were compared with those of the comparison groups using standardized differences.

A conditional logistic regression model was applied to assess the crude and adjusted odds ratios (ORs) and 95% Cis for gout and thyroid cancer. In the adjusted model, obesity, smoking, alcohol consumption, systolic blood pressure, diastolic blood pressure, fasting blood glucose, total cholesterol, and the CCI scores were adjusted with a stratification according to age, sex, income, and region of residence.

The participants were classified according to age (<60 years old; ≥60 years old), sex (males; females), income (low income; high income), region (urban; rural), obesity (underweight; normal weight; overweight; obese), smoking (non-smoker; former or current smoker), alcohol consumption (alcohol consumption < once a week; alcohol consumption ≥once a week), total cholesterol (<200 mg/dL; ≥200 to <240 mg/dL; ≥240 mg/dL), blood pressure (SBP < 140 mmHg and DBP < 90 mmHg; SBP ≥ 140 mmHg or DBP ≥ 90 mmHg), fasting blood glucose (<100 mg/dL; ≥100 mg/dL), and CCI score (score 0; score 1; score ≥ 2). The subgroups were analyzed in crude and adjusted models using an unconditional logistic regression model.

All analyses were two-tailed. A *p*-value < 0.05 was considered to be statistically significant. SAS version 9.4 (SAS Institute Inc., Cary, NC, USA) was used for the analyses.

## 3. Results

A total of 2.0% (116/5844) of the thyroid cancer group and 1.5% (354/23,376) of the comparison group had histories of gout (standardized difference (SD) = 0.04; Table 1). The thyroid cancer group demonstrated lower total cholesterol and fasting blood glucose levels and higher SBP and DBP than the comparison group. The rates of obesity, smoking status, alcohol consumption, and CCI score were distinct between the thyroid cancer and comparison groups.

The thyroid cancer group presented 1.32-fold higher odds of gout in the crude model (95% CI = 1.07–1.64, *p* = 0.010; Table 2). However, the association of thyroid cancer with gout was not maintained in the adjusted model (adjusted OR (aOR) = 1.24, 95% CI = 0.99–1.54, *p* = 0.062). Regarding age groups, the <60 years old age group showed higher odds of thyroid cancer in patients with a history of gout in the adjusted model (aOR = 1.36, 95% CI = 1.01–1.82, *p* = 0.041).

The normal weight group, abnormal blood pressure group, and CCI score = 0 groups also demonstrated higher odds of thyroid cancer in patients with a history of gout (aOR = 1.73, 95% CI = 1.13–2.66, *p* = 0.013 for the normal weight group; aOR = 1.37, 95% CI = 1.07–1.76, *p* = 0.014 for the abnormal blood pressure group; aOR = 1.48, 95% CI = 1.12–1.95, *p* = 0.005 for the CCI score = 0 group; Table 3). The other subgroups did not present a relationship of thyroid cancer with gout.

## 4. Discussion

Thyroid cancer was not associated with a previous history of gout in the total population. However, certain groups—such as patients < 60 years old, patients with a normal weight, patients with abnormal blood pressure, and patients without comorbidities—showed a positive association of thyroid cancer with a prior history of gout. When we searched the PubMed and EMBASE databases using the keywords “(thyroid cancer) AND (gout)” until March 2022, no study addressed the association between gout and thyroid cancer.

The presence of gout history has been suggested to elevate the risk of thyroid diseases in prior studies. In a cross-sectional study, female patients with gout demonstrated higher odds of hypothyroidism (OR = 2.44, 95% CI = 1.15–5.17) and Hashimoto’s thyroiditis (OR = 3.15, 95% CI = 1.53–6.49) [10]. Hyperthyroidism was associated with increased odds for gout in both males (OR = 1.37, 95% CI = 1.10–1.69) and females (OR = 2.13, 95% CI = 1.58–2.87) [16]. It was postulated that thyroid hormones influenced the kidney function through immune modulations [17]. Indirectly, a metabolic and cardiovascular compromise in thyroid dysfunction was suggested to increase urate levels by diminishing the renal function [17]. On the other hand, a retrospective study reported no evident link of thyroid disorders such as hypothyroidism and hyperthyroidism with gout [18]. Potential confounding effects and different study designs may have caused these heterogeneous results regarding the relationship between thyroid diseases and gout.

In the overall population, a previous history of gout was not related to the occurrence of thyroid cancer in this study. This lack of a significant association between thyroid cancer and pre-existing gout history could partially originate from the contribution of multiple risk factors to the development of thyroid cancer in addition to gout such as metabolic syndrome and cardiovascular diseases [19]. On the other hand, a few individuals with a history of gout in the middle-aged adult group (<60 years old) and the healthy patient group, which included those with a normal weight and without past medical histories, demonstrated a higher risk of thyroid cancer in our present study. In these groups of patients, the possible confounding or mediating effects of comorbid conditions on the occurrence of thyroid cancer may have been weaker than in those patients with multiple comorbid conditions. In addition, the impact of thyroid dysfunction on the development of thyroid cancer may have been more robust in the middle-aged population, which is the age group in which thyroid cancer is the most prevalent (median age of 51 years) [20]. Compared with other cancers, thyroid cancer is diagnosed at a younger age. For instance, the median age of diagnosis is approximately 62 years old for breast cancer and approximately 71 years old for lung cancer [20]. A thyroid cancer diagnosis at a young age is attributed to prior thyroid dysfunction in addition to possible overdiagnosis issues [21]. For instance, congenital hypothyroidism and maternal hypothyroidism, hyperthyroidism, and goiter were associated with an 18.12-fold increased risk of thyroid cancer in people of a young age (0–48 years old) [20]. Thus, the potential impact of thyroid dysfunction on the development of thyroid cancer cannot be discarded in these populations.

This study has improved previous knowledge on the contribution of gout to thyroid diseases by adding evidence of the relationship of gout with thyroid cancer. A large representative population was used and a comparison population was selected that was paired for age, sex, income, and region of residence. Multiple confounders were adjusted for in the association of previous gout with the subsequent occurrence of thyroid cancer. Moreover, subgroup analyses were performed to pinpoint a possible vulnerable group of gout patients who were exposed to the risk of thyroid cancer. However, a few limitations should be mentioned when interpreting the current results. For the diagnosis of gout, the severity and treatment histories—including medications—could not be considered in this study. The association of gout with thyroid cancer could be different according to the types or severity of the gout. For thyroid cancer, the histological subtypes and staging of diseases could not be accounted for in the present study. A previous study reported the association of hyperthyroidism and thyroiditis with differentiated thyroid cancer [6]. As gout has been reported to be associated with both hyperthyroidism and thyroiditis, the association of gout with thyroid cancer may be stronger in differentiated thyroid cancer than in undifferentiated thyroid cancer. In addition, although the comprehensive list of covariates was adjusted for in the current analyses, confounding factors may have remained such as a family history of thyroid cancer [3]. Future studies with classified disease categorizations for gout and thyroid cancer can address these issues.

## 5. Conclusions

A prior history of gout did not increase the risk of a subsequent diagnosis of thyroid cancer. However, middle-aged individuals with a history of gout and healthy individuals without comorbidities with a history of gout presented a higher risk of thyroid cancer. The potential risk of thyroid cancer in these patients with gout needs to be considered in clinics.

## Figures and Tables

**Table 1:** General characteristics of participants.

Characteristics	Total Participants		
	Thyroid Cancer (N = 5844)	Comparison (N = 23,376)	Standardized Difference
Age (years old, *n*, %)			0.00
40–44	127 (2.2)	508 (2.2)	
45–49	837 (14.3)	3348 (14.3)	
50–54	1593 (27.3)	6372 (27.3)	
55–59	1344 (23.0)	5376 (23.0)	
60–64	896 (15.3)	3584 (15.3)	
65–69	592 (10.1)	2368 (10.1)	
70–74	325 (5.6)	1300 (5.6)	
75–79	102 (1.8)	408 (1.8)	
80–84	27 (0.5)	108 (0.5)	
≥85	1 (0.0)	4 (0.0)	
Sex (*n*, %)			0.00
Male	1234 (21.1)	4936 (21.1)	
Female	4610 (78.9)	18,440 (78.9)	
Income (*n*, %)			0.00
1 (lowest)	732 (12.5)	2928 (12.5)	
2	683 (11.7)	2732 (11.7)	
3	901 (15.4)	3604 (15.4)	
4	1195 (20.5)	4780 (20.5)	
5 (highest)	2333 (39.9)	9332 (39.9)	
Region of residence (*n*, %)			0.00
Urban	2802 (48.0)	11,208 (48.0)	
Rural	3042 (52.1)	12,168 (52.1)	
Total cholesterol level (mg/dL, mean, SD)	198.7 (38.0)	201.5 (37.7)	0.08
SBP (mmHg, mean, SD)	124.4 (15.9)	123.5 (16.1)	0.05
DBP (mmHg, mean, SD)	77.5 (10.5)	76.7 (10.5)	0.08
Fasting blood glucose level (mg/dL, mean, SD)	97.2 (23.1)	98.2 (26.1)	0.04
Obesity * (*n*, %)			0.11
Underweight	79 (1.4)	453 (1.9)	
Normal	1936 (33.1)	8772 (37.5)	
Overweight	1654 (28.3)	6370 (27.3)	
Obese I	1936 (33.1)	6998 (29.9)	
Obese II	239 (4.1)	783 (3.4)	
Smoking status (*n*, %)			0.08
Non-smoker	5113 (87.5)	20,053 (85.8)	
Past smoker	392 (6.7)	1474 (6.3)	
Current smoker	339 (5.8)	1849 (7.9)	
Alcohol consumption (*n*, %)			0.01
<1 time a week	4576 (78.3)	18,171 (77.7)	
≥1 time a week	1268 (21.7)	5205 (22.3)	
CCI score † (score, *n*, %)			0.41
0	3528 (60.4)	18,201 (77.9)	
1	941 (16.1)	2840 (12.2)	
≥2	1375 (23.5)	2335 (10.0)	
Gout (*n*, %)	116 (2.0)	354 (1.5)	0.04

CCI, Charlson Comorbidity Index; DBP, diastolic blood pressure; SBP, systolic blood pressure; SD, standard deviation. * Obesity (BMI, body mass index, kg/m^2^) was categorized as <18.5 (underweight), ≥18.5 to <23 (normal), ≥23 to <25 (overweight), ≥25 to <30 (obese I), and ≥30 (obese II). † Thyroid cancer was excluded in the CI score.

**Table 2:** Crude and adjusted odds ratio (95% confidence interval) of gout for thyroid cancer with subgroup analyses according to age, sex, income, and region of residence.

Characteristics	Thyroid Cancer	Comparison	OR (95% CIs) for Thyroid Cancer			
	(Exposure/Total, %)	(Exposure/Total, %)	Crude †	*p*-Value	Adjusted † ‡	*p*-Value
Total participants (*n* = 29,220)	116/5844 (2.0)	354/23,376 (1.5)	1.32 (1.07–1.64)	0.010 *	1.24 (0.99–1.54)	0.062
Age						
Age < 60 years old (*n* = 19,505)	67/3901 (1.7)	187/15,604 (1.2)	1.45 (1.09–1.92)	0.011 *	1.36 (1.01–1.82)	0.041 *
Age 60 years old (*n* = 9715)	49/1943 (2.5)	167/7772 (2.2)	1.18 (0.85–1.64)	0.314	1.11 (0.79–1.56)	0.532
Sex						
Males (*n* = 6170)	61/1234 (4.9)	201/4936 (4.1)	1.23 (0.91–1.65)	0.174	1.15 (0.85–1.56)	0.377
Females (*n* = 23,050)	55/4610 (1.2)	153/18,440 (0.8)	1.44 (1.06–1.97)	0.020 *	1.36 (0.99–1.87)	0.062
Income						
Low income (*n* = 11,580)	48/2316 (2.1)	126/9264 (1.4)	1.54 (1.10–2.17)	0.012 *	1.39 (0.98–1.98)	0.067
High income (*n* = 17,640)	68/3528 (1.9)	228/14,112 (1.6)	1.20 (0.91–1.58)	0.194	1.14 (0.86–1.52)	0.375
Region of residence						
Urban (*n* = 14,010)	47/2802 (1.7)	155/11,208 (1.4)	1.22 (0.88–1.70)	0.240	1.12 (0.79–1.59)	0.522
Rural (*n* = 15,210)	69/3042 (2.3)	199/12,168 (1.6)	1.41 (1.06–1.86)	0.017 *	1.32 (0.99–1.76)	0.059

CCI, Charlson Comorbidity Index; CI, confidence interval; DBP, diastolic blood pressure; OR, odds ratio; SBP, systolic blood pressure. * Conditional logistic regression, significance at *p* < 0.05. † Models were stratified by age, sex, income, and region of residence. ‡ Adjusted for total cholesterol, SBP, DBP, fasting blood glucose, obesity, smoking, alcohol consumption, and CCI scores.

**Table 3:** Subgroup analyses regarding odds ratio (95% confidence interval) of gout for thyroid cancer with subgroup analyses according to obesity, smoking status, alcohol consumption, total cholesterol, blood pressure, fasting blood glucose, and CCI scores.

Characteristics	Thyroid Cancer	Comparison	OR (95% CIs) for Thyroid Cancer			
	(Exposure/ Total, %)	(Exposure/Total, %)	Crude	*p*-Value	Adjusted †	*p*-Value
Obesity						
Underweight (*n* = 532)	2/79 (2.5)	2/453 (0.4)	5.86 (0.81–42.25)	0.079	6.70 (0.80–56.09)	0.079
Normal weight (*n* = 10,708)	32/1936 (1.7)	83/8772 (1.0)	1.76 (1.17–2.65)	0.007 *	1.73 (1.13–2.66)	0.013 *
Overweight (*n* = 8024)	26/1654 (1.6)	100/6370 (1.6)	1.00 (0.65–1.55)	1.000	1.03 (0.66–1.61)	0.891
Obese (*n* = 9956)	56/2175 (2.6)	169/7781 (2.2)	1.19 (0.88–1.62)	0.264	1.08 (0.79–1.49)	0.621
Smoking status						
Non-smoker (*n* = 25,166)	84/5113 (1.6)	249/20,053 (1.2)	1.33 (1.04–1.71)	0.025 *	1.20 (0.93–1.56)	0.170
Past or current smoker (*n* = 4054)	32/731 (4.4)	105/3323 (3.2)	1.40 (0.94–2.10)	0.100	1.38 (0.91–2.10)	0.132
Alcohol consumption						
<1 time a week (*n* = 22,747)	75/4576 (1.6)	222/18171 (1.2)	1.35 (1.04–1.75)	0.027 *	1.23 (0.93–1.62)	0.144
≥1 time a week (*n* = 6473)	41/1268 (3.2)	132/5205 (2.5)	1.28 (0.90–1.83)	0.168	1.22 (0.84–1.78)	0.290
Total cholesterol (mg/dL)						
<200 (*n* = 14,984)	67/3160 (2.1)	197/11,824 (1.7)	1.28 (0.97–1.69)	0.085	1.28 (0.95–1.72)	0.100
≥200 to < 240 (*n* = 10,041)	34/1893 (1.8)	105/8148 (1.3)	1.40 (0.95–2.07)	0.090	1.33 (0.89–1.99)	0.161
≥240 (n = 4195)	15/791 (1.9)	52/3404 (1.5)	1.25 (0.70–2.23)	0.457	0.96 (0.52–1.78)	0.898
Blood pressure (mmHg)						
SBP < 140 and DBP < 90 (*n* = 23,458)	89/4655 (1.9)	256/18,803 (1.4)	1.41 (1.11–1.80)	0.005 *	1.37 (1.07–1.76)	0.014 *
SBP ≥ 140 or DBP ≥ 90 (*n* = 5762)	27/1189 (2.3)	98/4573 (2.1)	1.06 (0.69–1.63)	0.784	0.92 (0.58–1.45)	0.709
Fasting blood glucose (mg/dL)						
<100 (*n* = 19,981)	75/4040 (1.9)	216/15,941 (1.4)	1.38 (1.06–1.80)	0.018 *	1.26 (0.95–1.66)	0.106
≥100 (*n* = 9239)	41/1804 (2.3)	138/7435 (1.9)	1.23 (0.86–1.75)	0.250	1.18 (0.81–1.70)	0.389
CCI score						
0 (*n* = 21,729)	68/3528 (1.9)	233/18,201 (1.3)	1.52 (1.16–1.99)	0.003 *	1.48 (1.12–1.95)	0.005 *
1 (*n* = 3781)	19/941 (2.0)	47/2840 (1.7)	1.23 (0.72–2.10)	0.460	1.19 (0.69–2.05)	0.544
≥2 (*n* = 3710)	29/1375 (2.1)	74/2335 (3.2)	0.66 (0.43–1.02)	0.060	0.85 (0.53–1.36)	0.504

CCI, Charlson Comorbidity Index; CI, confidence interval; DBP, diastolic blood pressure; OR, odds ratio; SBP, systolic blood pressure. * Unconditional logistic regression, significance at *p* < 0.05. † Adjusted for age, sex, income, region of residence, total cholesterol, SBP, DBP, fasting blood glucose, obesity, smoking, alcohol consumption, and CCI scores.

## Data Availability

Restrictions apply to the availability of these data. Data were obtained from the Korean National Health Insurance Sharing Service (NHISS) and are available at https://nhiss.nhis.or.kr (accessed on 25 January 2022) with the permission of the NHISS.
